# Role of Proteases in Chronic Obstructive Pulmonary Disease

**DOI:** 10.3389/fphar.2017.00512

**Published:** 2017-08-08

**Authors:** Kailash C. Pandey, Sajal De, Pradyumna K. Mishra

**Affiliations:** ^1^Department of Biochemistry, National Institute for Research in Environmental Health (ICMR) Bhopal, India; ^2^Department of Pulmonary Medicine, National Institute for Research in Environmental Health (ICMR) Bhopal, India; ^3^Department of Molecular Biology, National Institute for Research in Environmental Health (ICMR) Bhopal, India

**Keywords:** COPD, cysteine protease, mettaloproteinase, degradation, oxidative stress, apoptosis, caspase, protease-antiprotease imbalance

## Abstract

Chronic obstructive pulmonary disease (COPD) is generally associated with progressive destruction of airways and lung parenchyma. Various factors play an important role in the development and progression of COPD, like imbalance of proteases, environmental and genetic factors and oxidative stress. This review is specifically focused on the role of proteases and their imbalance in COPD. There are three classes (serine, mettalo, and cysteine) of proteases involved in COPD. In serine proteases, neutrophil elastase, cathepsin G, and proteinase-3 are involved in destruction of alveolar tissue. Matrix-mettaloproteinase-9, 12, 13, plays an influential role in severity of COPD. Among cysteine proteases, caspase-3, caspases-8 and caspase-9 play an important role in controlling apoptosis. These proteases activities can be regulated by inhibitors like α-1-antitrypsin, neutrophil elastase inhibitor, and leukocyte protease inhibitor. Studies suggest that neutrophil elastase may be a therapeutic target for COPD, and specific inhibitor against this enzyme has potential role to control the disease. Current study suggests that Dipeptidyl Peptidase IV is a potential marker for COPD. Since the expression of proteases and its inhibitors play an important role in COPD pathogenesis, therefore, it is worth investigating the role of proteases and their regulation. Understanding the biochemical basis of COPD pathogenesis using advanced tools in protease biochemistry and aiming toward translational research from bench-to-bedside will have great impact to deal with this health problem.

## General introduction and pathology

**Figure 1:**
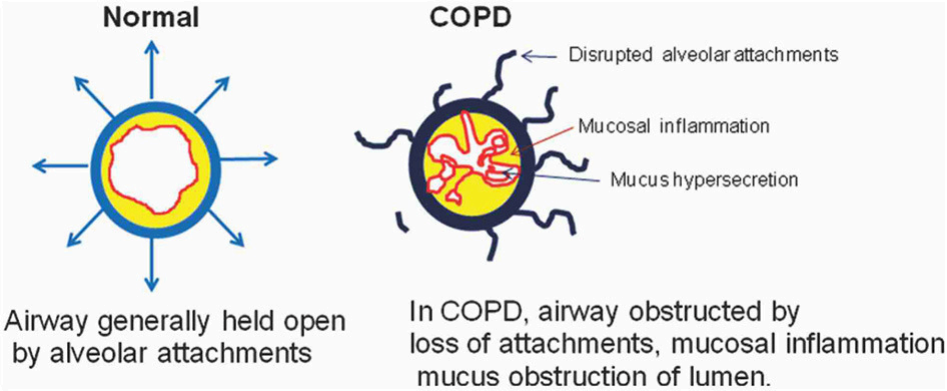
Air flow in Normal vs. COPD. The airway in normal condition is distended by alveolar attachments at the time of expiration. But in COPD these alveolar attachments are mainly disrupted due to emphysema, therefore contributing to airway closure during expiration, and hyperinflation due to air trap in the alveoli. Inflammation, fibrosis and mucus secretions further obstructed and distorted the peripheral airways and consequently create the poor mucociliary clearance (Barnes, 2004).

Chronic obstructive pulmonary disease (COPD) is enhanced inflammatory response of airways as well as lung parenchyma to harmful particles or gases. In COPD, airways are obstructed by loss of alveolar attachments, mucosal inflammation, and mucous obstruction of lumen (Figure 1).

The inflammation causes structural damage, narrowing of airways, and the destruction of lung parenchyma (emphysema) (Demedts et al., 2006). The airflow obstruction in COPD is not fully reversible and usually progressive. Besides inflammation, the other processes involved in the pathogenesis of COPD are (i) an imbalance between proteases and anti-proteases, (ii) imbalance between oxidants and antioxidants (Demedts et al., 2006). COPD broadly encompasses two pathologic entities i.e., emphysema and chronic bronchitis. Emphysema is defined by alveolar wall destruction and irreversible enlargement of the air spaces distal to the terminal bronchioles and without evidence of fibrosis (Murray and Lopez, 1999). These components vary in proportion among COPD patients. A clinical diagnosis of COPD is considered in any patient with dyspnea, chronic cough, sputum production as well as history of exposure to the disease. As defined by the National Institute for Clinical Excellence (NICE), a diagnosis of COPD is based on the ratio of forced expiratory volume/second (FEV1)/ forced vital capacity (FVC) is <0.7 in spirometry analysis suspect the presence of airflow limitation and thus of COPD (Murray and Lopez, 1999). Unfortunately, COPD is undiagnosed until there are clinical symptoms and the disease is moderately advanced, therefore a mild condition may go unnoticed in the community. It is estimated that about 3 million deaths were caused by this disease in 2015, globally. A current epidemiological estimation predicted by 2020, COPD may become the third leading cause of death worldwide (Murray and Lopez, 1999; Peto et al., 1999). A major risk factor for the development of COPD is tobacco smoking (Peto et al., 1999; Vestbo et al., 2014). Other risk factors are air pollution (both indoor and outdoor), occupation exposure to dust and chemicals, and frequent respiratory tract infections in childhood. There are available drugs for COPD based on anti-cholinergics, beta-2-agonist, phosphodiesterase-4-inhibitors and corticosteroids (Prakash et al., 2013). Despite intensive research all over the world, the biochemical, cellular, and molecular mechanisms underlying COPD pathobiology are not yet well understood. Therefore, the scientific community needs to investigate this important health problem with modern tools.

An important question is the identification of factors which interact with smoking and causes more rapid decline in FEV1 as well as the development of COPD. These may be caused by either additional environmental factors or susceptibility of genes (Murray and Lopez, 1999). Analyzing these factors into detail will provide new avenues to prevent the development of this disease and target potential pathways that can reduce disease progression. Published literature regarding sputum proteomic analysis suggests that changes in various proteins are associated with the development of Acute Exacerbation COPD (AECOPD), caused by heavy smoking (Uh et al., 2012; Faner et al., 2014). A serum surfactant protein D (SP-D) has been suggested as a marker in patients with AECOPD, but unfortunately cannot distinguish between COPD and AECOPD (Lomas et al., 2009; Koutsokera et al., 2013). A recent study suggested that microfibrillar-associated protein 4 (MFAP4), a matricellular glycoprotein that co-localizes with elastic fibers expressed in the lung tissues, is associated with the severity in COPD (Kishore et al., 2006; Johansson et al., 2014). Other marker, a C-reactive protein (CRP) binds to bacteria, oxidized lipids, and apoptotic cells, consequently facilitates their clearance via innate immune system. Therefore, act as an inflammatory marker for heart and lung diseases, including COPD, but it is not specific marker for COPD, and its role in COPD is not yet established (Bircan et al., 2008). Among environmental factors, tobacco smoking is the most important risk factor for the development of COPD (Peto et al., 1999). Oxidative stress is another factor contributes to COPD, generally occurs when reactive oxygen species are produced in excess (Barnes, 2004). The genetic variations (deficiencies of α-antitrypsin) have also been linked to COPD associated pathogenesis (Perlmutter, 1996). This review will specifically focus on the role of proteases in COPD.

## Indian prospective

The prevalence of COPD varies from 3–8% among Indian males and approximately 2.5–4.5% in Indian females (Bhome, 2012). It is estimated that by the end of 2016, more than 5.7 million people are suffering from COPD in India, and the estimated economic burden of COPD (2010–2011) was more than six billion USD (Kalkana et al., 2016). The Indian study on epidemiology of asthma, respiratory symptoms and chronic bronchitis in adults involved a total of 85,105 men, 84,470 women from 12 urban and 11 rural sites (Jindal et al., 2012). This study has shown that the overall prevalence of chronic bronchitis in adults >35 years was around 3.5% (Jindal et al., 2012). There are wide variations in the occurrence of COPD in Indian subcontinent (Kalkana et al., 2016). Based on these studies, the national burden of chronic bronchitis was estimated around 14 to 14.5 million (Jindal et al., 2012; Salvi and Agarwal, 2012; Vijayan, 2013). A long term epidemiological study on the health effect of toxic gas in Bhopal, was carried out by ICMR-National Institute for Research in Environmental Health, revealed that the highest percentage (22%) of people in the toxic gas exposed cohort suffered from various respiratory morbidities including COPD (NIREH, 2014).

Previously a multicentric study based on epidemiology of COPD and its relationship with tobacco smoking and environmental tobacco smoke exposure was studied (Jindal et al., 2006). A total 35295 subjects were included in this study, 4.1% COPD cases diagnosed with male and female ratio of 1.5: 1, and smoker to non- smoker ratio of 2.6:1. This study also indentified the prevalence among Bidi (filled with crude tobacco flake and mainly wrapped in a *Piliostigma racemosum* leaf) and cigarette smokers was 8.2 and 5.9%, respectively (Jindal et al., 2006). A recent cross sectional study was conducted among twelve hundred adults in Delhi, India (Sinha et al., 2017). This study suggested the prevalence of COPD was 10%, and the tobacco smoking adults were the strongest risk factor with this disease. The old smoker had 63 % lesser risk as compare to current smokers (Sinha et al., 2017). Further, environmental smoke, occupational exposure, age factor, and biomass fuel are the others important factors which influence this health problem (Sinha et al., 2017). Due to heterogeneity, limited numbers of studies and further their unsuitability for meta-analysis, these discussed figures are most unlikely to apply for all subpopulations in India. Therefore, the general prevalence of COPD from all across the country largely remains unknown.

### Role of proteases and their association with COPD

**Figure 2:**
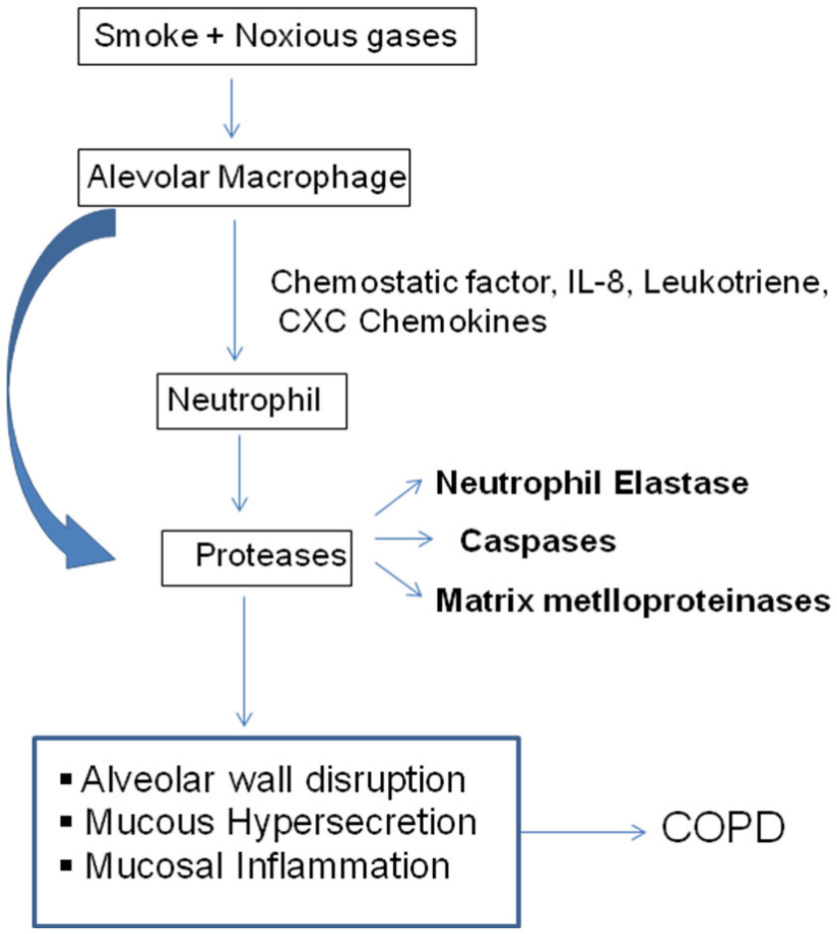
Inflammatory mechanism in COPD. Cigarette smoke or other toxic particles activates the macrophage in the respiratory tract and release neutrophil chemotactic factors like IL-8, leukotrine, CXC chemokines. These cells further release proteases of different classes, which break down connective tissue in the lung parenchyma resulting COPD (Barnes, 2004).

Proteases cleave proteins into smaller fragments and classified according to their catalytic site. Proteases associated with COPD pathology has been divided into three main classes; serine protease, matrix-mettaloproteinase, cysteine protease (Figure 2; Barnes, 2004).

Proteases are involved in pathogenesis of various diseases such as arthritis, osteoporosis, AIDS, immune-related diseases, atherosclerosis, cancer, and for a wide variety of parasitic diseases e.g., malaria, amebiasis, chagas disease, leishmaniasis, or African sleeping sickness, therefore act as potential targets (Lecaille et al., 2002; Pandey et al., 2005; Gills et al., 2007; Salminen-Mankonen et al., 2007; Verma et al., 2016). Targeting proteases in COPD still need to be explored in great detail.

### Role of serine protease in COPD

Seine proteases (or serine endopeptidases) belongs to a PA clan and S1 (trypsin/chymotrypsin) family of proteolytic enzymes. S1 family include neutrophil elastase (NE), protinase-3, cathepsin G. Studies suggest that these enzymes are synthesized as pro-enzymes in the ER and further processed by cleavage of the signal peptide and finally removal of a dipeptide by cathepsin C (Belaaouaj et al., 1998). Serine proteases have been reported to be located in blood monocytes, mast cells and neutrophils, act as potent mucus stimulants (Qiu et al., 2003). Neutrophil elastase, a serine proteases play as potent secretor, therefore mucus might aggravate airflow obstruction in COPD (Lee et al., 2015). This enzyme is mainly involved in the destruction of alveolar tissue (Qiu et al., 2003). The mice model study suggested that elastase play important role in emphysema (Belaaouaj et al., 1998), further study suggest that NE has a role in pathogenesis of COPD by enhancing inflammation and apoptosis (Belaaouaj et al., 1998; Qiu et al., 2003). NE is released by activated neutrophils and macrophages, which induce small airway and alveolar epithelial cell apoptosis using intrinsic pathway (Lee et al., 2015). These reactions slow down a serine/threonine protein kinase phosphorylation and activate proteinase activated receptor-1 (PAR) and finally proceed for apoptosis pathway by caspase-3(Qiu et al., 2003; Lee et al., 2015). The potent NE inhibitors with nM ranges of IC_50_, have been identified (Tsai and Hwang, 2015). These inhibitors including, pyrimidinone, tetra-hydro-pyrrolo-pyrimidinedione, pyrazinone, benzoxazinone, and uracil derivatives have been indentified (Tsai and Hwang, 2015). Using templates of these existing inhibitors and detail kinetic study of NE, we believe that in the future more potential compounds with efficient therapeutic NE inhibitors will become available for clinical applications. Some of the potential drugs against NE will be discussed in the section entitled targeting neutrophil elastase.

## Role of dipeptidyl peptidase IV

Dipeptidyl Peptidase IV **(**DPPIV) is a serine expopeptide that catalyzes the release of an N-terminal dipeptide. DPPIV is present as a homo dimer on the cell surface and catalytically active protease. DPPIV is a multifunctional cell surface protein express in cell types including T lymphocytes (Javidroozi et al., 2012). DPPIV present in the serum as soluble truncated form of enzyme without transmenbrane and intracellular domains. It has already been established that DPPIV plays an important role in tumor biology as a suppressor in the development of cancer, therefore a useful biomarker for different cancers (Schade et al., 2008; Cordero et al., 2009; Javidroozi et al., 2012; Larrinaga et al., 2012). In addition, DPPIV also plays an important role in glucose metabolism, e.g., DPPIV is responsible for the degradation of glucagon-like peptide-1 (Somborac-Bačura et al., 2012). But interestingly, DPPIV also plays an important role in the inflammatory response in the lungs, therefore it is worth to mention with related to lung biology (Somborac-Bačura et al., 2012). In fact, recently, it has been shown that the serum containing DPPIV activity in patients with stable COPD (722–1,037 ng/ml) is significantly lower than healthy people (1,080–1,620 ng/ml) (Holst and Deacon, 2005). Further, the level of this enzyme is not affected by smoking, age, and other factors (Holst and Deacon, 2005). Study systematically investigated the correlation between serum level of DPPIV and types of COPD, including stable COPD, AECOPD, measuring serum concentrations of DPPIV with ELISA, followed by a comprehensive analysis of the relationships between a set of parameters of COPD and different forms of COPD using statistical analysis methods (Holst and Deacon, 2005; Chang et al., 2016). The above mentioned study suggested that DPPIV was signifintely decreased in both stable COPD (722–1,037 ng/ml) and AECOPD (610–966 ng/ml) compare to healthy people (1,080–1,620 ng/ml). Therefore, DPPIV may be a predictor in patients having COPD and seems to be a valuable serologic biomarker. However, detail study with larger number of patients and the role of this enzyme in COPD are indeed required to further strengthen the preliminary data.

### Role of matrix mettaloproteinase in COPD

Matrix mettaloproteinase (MMPs) are a family of matrix degrading enzymes which degenerate the protein components of extracellular matrix (Finlay et al., 1997). MMPs are believed to be important for the development, tissue remodeling and repairing of damaged tissue. MMPs are produced by inflammatory cells e.g., neutrophils and alveolar macrophage (Finlay et al., 1997). The MMPs are divided into different subgroups according to their primary substrates like collagenases which include MMP-1 and MMP-13. Using MALDI-TOF MS analysis, Lee et al., showed that the expression of matrix metalloproteinase-13 (MMP-13) was increased in lung tissues of COPD patients (Lee et al., 2009). MMP-13, a major proteolytic enzyme believes to be implicated in tissue damage and remodeling, which is predominantly expressed in alveolar macrophages and type II pneumocytes (Chaudhuri et al., 2012). Presence of MMP-12 in sputum and activity in patients with COPD are directly associated with the extent of emphysema measured by means of lung function and computed tomography (Chaudhuri et al., 2012). Further Haq et al. suggested that a single nucleotide polymorphisms reduce the enzyme activity of MMP-12 and consequently protect from emphysema in COPD (Haq et al., 2010, 2011). The genetic and animal studies have suggested MMP-12 in the pathogenesis of COPD (Haq et al., 2010, 2011). MMP-12 SNP affects the enzyme activity, and protects against emphysema in COPD (Jormsjo et al., 2000; Haq et al., 2010). It has previously been shown that individuals with homozygous for the A/A allele of rs652438 in MMP-12, are prone to suffer with severe COPD (Jormsjo et al., 2000; Haq et al., 2010). Study also implicated that rs652438 SNP changes the MMP-12 activity and increased macrophage infiltration and emphysema in the lungs of COPD patients (Jormsjo et al., 2000; Haq et al., 2011).

MMP-2 and MMP-9 mainly degrade basal membrane components like fibronectin; elastin, and these proteases have a broad spectrum of extra cellular membrane as targets. MMP-9 plays an important role in COPD, it is 92–96 kD in size, the sources of MMP-9 have been keratinocytes, monocytes, leukocytes, macrophages, and fibroblasts (Emjabbar et al., 1999; Johnson, 2016). It plays an important role in the cellular invasion of the basement membrane by mononuclear phagocytes, synovial fibroblasts. MMP-9 has been considered to play a major role in cell migration and airway inflammatory response in COPD. Studies including *in vitro* and *in vivo* suggested that MMP-9 may play an important role during eosinophil migration and influence the severity of COPD (Van den Steen et al., 2002; Linder et al., 2015). A population-based cross-sectional study demonstrated that in serum of COPD, MMP-9 concentration is higher compare to non-COPD, and production of cough and decreasing FEV1 were associated with MMP-9 in COPD (Legrand et al., 1999; Linder et al., 2015). An increase of ~10 to 100 folds concentrations of MMP-9 and MMP-3 in the epithelial lining fluid of the patients with respiratory problem suggests that they may involved in COPD (Legrand et al., 1999). MMP-9 controls the migration of repairing human bronchial epithelial cells by remodeling the provisional extracellular matrix, remodeling is facilitates by wound healing, angiogenesis, establishment of more stable fibronectin-containing contacts in the central part of the cell (Legrand et al., 1999; Linder et al., 2015). MMP-9 (or Type IV collagenase) is abundant in various lung diseases e.g.. asthma, idiopathic pulmonary fibrosis, and COPD, while their levels are reportedly low in normal human lungs. Its role in matrix remodeling makes it an important candidate during fibrosis that occurs in COPD.

### Role of cysteine proteases

**Figure 3:**
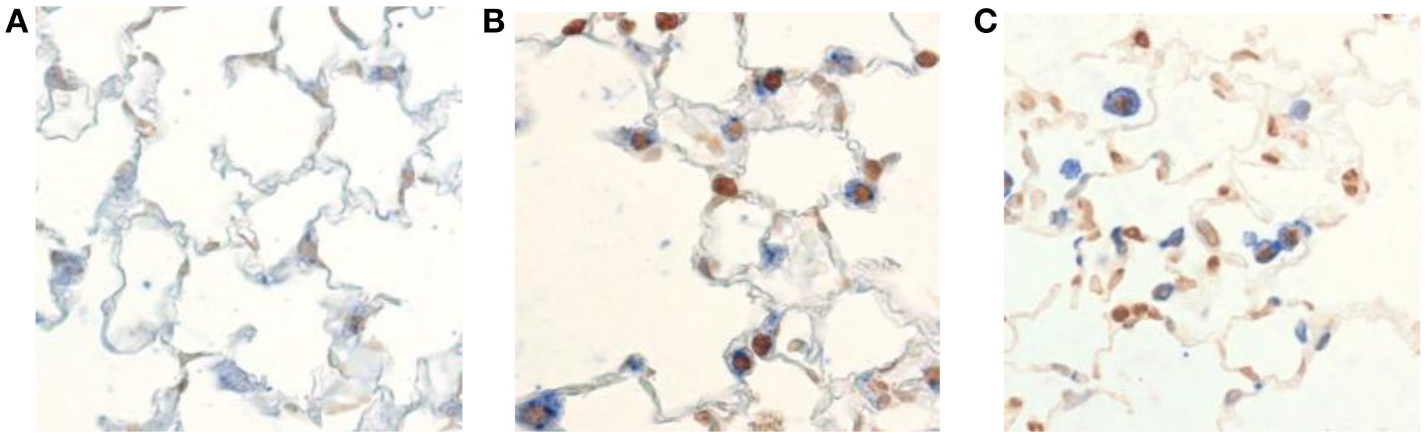
Visualization of apoptotic cells in the lung of mice with COPD. TUNEL-staining demonstrates an increase in apoptotic cells (dark brown nuclei) in the lungs of mice exposed to cigarette smoke **(B,C)** compared to air-exposed animals **(A)**. This figure has been adapted from Demedts et al. (2006).

Increasing number of data suggests that cysteine proteases; caspase-3, caspases-8 and caspase-9 play important roles in COPD (Muzio et al., 1998; Yasuda et al., 1998; Demedts et al., 2006; Abboud and Vimalanathan, 2008). These caspases are involved in COPD by controlling the mechanism of apoptosis. The death of structural cells in the lung possibly is an important event in the pathogenesis of COPD (Hirata et al., 1998; Muzio et al., 1998). Published literature suggested that there is an increase in apoptotic alveolar epithelial and endothelial cells in the lungs of COPD patients (Hirata et al., 1998). The above mentioned caspases mRNA expression were higher in COPD cells (Hirata et al., 1998). This study suggests that in COPD, the apoptosis is mediated by the receptor mediated extrinsic pathway by activation of caspase-8 (Hirata et al., 1998). The enhanced p53 (cell cycle regulatory protein) levels can stimulate the induction of caspase-8 resulting in apoptosis of bronchial epithelial cell in COPD (Yasuda et al., 1998). Inflammatory response and oxidative stress signals in COPD, are mediated by binding of members of the tumor necrosis factor family (Fas ligand, TNF-α). Consequently, formation of the death induce signal complex (DISC), which is made up of multiple adaptor molecules such as the Fas associated death domain (FADD). Yasuda et al. also suggested a significant increase in death receptor (Fas) in the plasma of COPD patients (Yasuda et al., 1998). A death receptor recruits procaspase-8 and leads to the autolytic activation to caspase-8, and finally start apoptosis using caspase-3 (Hirata et al., 1998; Muzio et al., 1998; Yasuda et al., 1998). In summary, it is suggested that in case of COPD pathogenesis, caspase-9, caspase-8, caspase-3 play crucial role in apoptosis (Kidokoro et al., 1977; Perlmutter, 1996; Churg and Wright, 2005; Gogebakan et al., 2014). Further, using TUNEL-staining in lung cells of mice with COPD suggested that an increase in apoptotic cells compare to normal cells (Demedts et al., 2006) (Figure 3). Those mediators involved in apoptosis could be interesting targets to prevent the development of COPD. Therefore, it is important to explore the role of caspases in COPD.

## Protease-antiprotease imbalance in COPD pathogenesis

Published literature using human and animal studies support the evidence that protease-anti-proteases imbalance may play crucial role in the pathogenesis of COPD (Gadek and Pacht, 1990; Muzio et al., 1998; Churg and Wright, 2005; Demedts et al., 2006; Abboud and Vimalanathan, 2008; Gogebakan et al., 2014). Excess neutrophils accumulation and activation disrupt the protease-antiprotease balance and trigger lung destruction process in COPD (Churg and Wright, 2005; Gogebakan et al., 2014; Demedts et al., 2006). The endogenous secretory neutrophils elastase inhibitors neutralize the proteolytic activity of neutrophil elastase, and these inhibitors are abundant in the respiratory tract which controls the activity of neutrophil elastase (Kidokoro et al., 1977; Abboud and Vimalanathan, 2008). A protease inhibitor, α-1-antitrypsin plays an important role in controlling proteases activities. This inhibitor exerts potent control over the proteolytic activity of neutrophil elastase, proteinase-3, cathepsin G, and neutrophils serine protease-4 (Perlmutter, 1996). The deficiency of α-1-antitrypsin results in emphysema, which can be successfully treated via exogenous supplementation of α-1-antitrypsin (Perlmutter, 1996). Study suggests that the treatment of COPD patients with α-1-antitrypsin deficiency, reduce mortality and slow the progression of emphysema (Perlmutter, 1996). A molecule like secretory leukocyte protease inhibitor (SLPI) and elafin, members of the chelonianin family, are also able to inhibit NE and control the proteolytic activity (Tsai and Hwang, 2015). Since SLPI and elafin are primarily produced by respiratory tract epithelial cells, it seems they may play an important role in COPD.

The balance of protease-antiprotease may be affected by a low level of α-1-antitrypsin or insufficient production of α-1-antitrypsin because of genetic defects or the inactivation of α-1-antitrypsin by smoking-induced oxidants. The genetic variations (deficiencies of α-1-antitrypsin) have also been linked to COPD associated pathogenesis (Perlmutter, 1996). The deficiency of α-1-antitrypsin causes non-regulation and over expression of neutrophil elastase that result in lung parenchymal destruction. The critical role of neutrophil elastase in anti-trypsin-deficient COPD is supported by the correlation of increased leukocyte elastase concentration (Perlmutter, 1996). Beside gene deficiency, environmental toxin and smoking may cause a protease-anti-protease imbalance in the lung by reducing the functional activity of α-1-antitrypsin in the lung interstitium and “alveolar” lining fluid by increasing the amount of elastolytic proteases released in the lung (Perlmutter, 1996; Hirata et al., 1998; Demedts et al., 2006). Literature further suggested that oxidative stress also impair the function of anti-proteases such as α-1-antitrypsin and SLPI and thereby accelerates the breakdown of elastin in lung parenchyma (Perlmutter, 1996; Hirata et al., 1998). Since expression of proteases and its inhibitors play significant role in COPD pathogenesis, therefore, it is worth to investigate the role of proteases and their regulation.

## Targeting neutrophil elastase

In normal condition, neutrophil elastase express on the cellular membrane, and only 4–5% being released into the extracellular (Tsai and Hwang, 2015). But in COPD, neutrophil elastase is much more available extracellularly because of the increased concentration of necrotic neutrophils (Kidokoro et al., 1977). In COPD patients, the airway leads to neutrophil necrosis which promote to an excessive and uncontrolled secretion of enzyme (Kidokoro et al., 1977). The highest NE activity was found in BAL fluid of severe COPD patients (Kidokoro et al., 1977; Demedts et al., 2006), which can damage the tissue by degradation of TIMP-1 and activation of pro-MMP-9. In addition, NE is a very potent inducer of mucous gland hyperplasia, one of the characteristics of emphysema (Kidokoro et al., 1977). Excessive NE activity as well as indirect burden of this enzyme can be restricted by using specific inhibitors. Other approach is to decrease neutrophil accumulation by inhibiting dipeptide peptide activity (or cathepsin C), since cathepsin C post-translationally process inactive NE into an active enzyme (Demedts et al., 2006). This strategy indirectly decreased total NE activity and believes to be advantageous in COPD. The literature has increasingly implicated the role of NE in the pathogenesis of COPD, however only few inhibitors have been validated for therapeutic use in clinical settings. Sivelestat is the only NE synthetic inhibitor in clinical market currently approved in Japan and South Korea to treat systemic inflammatory response syndrome (Vandivier et al., 2002). There are some effective inhibitors against NE, which need to study in great detail.

## Inhibitor based on neutrophil elastase

**Figure 4:**
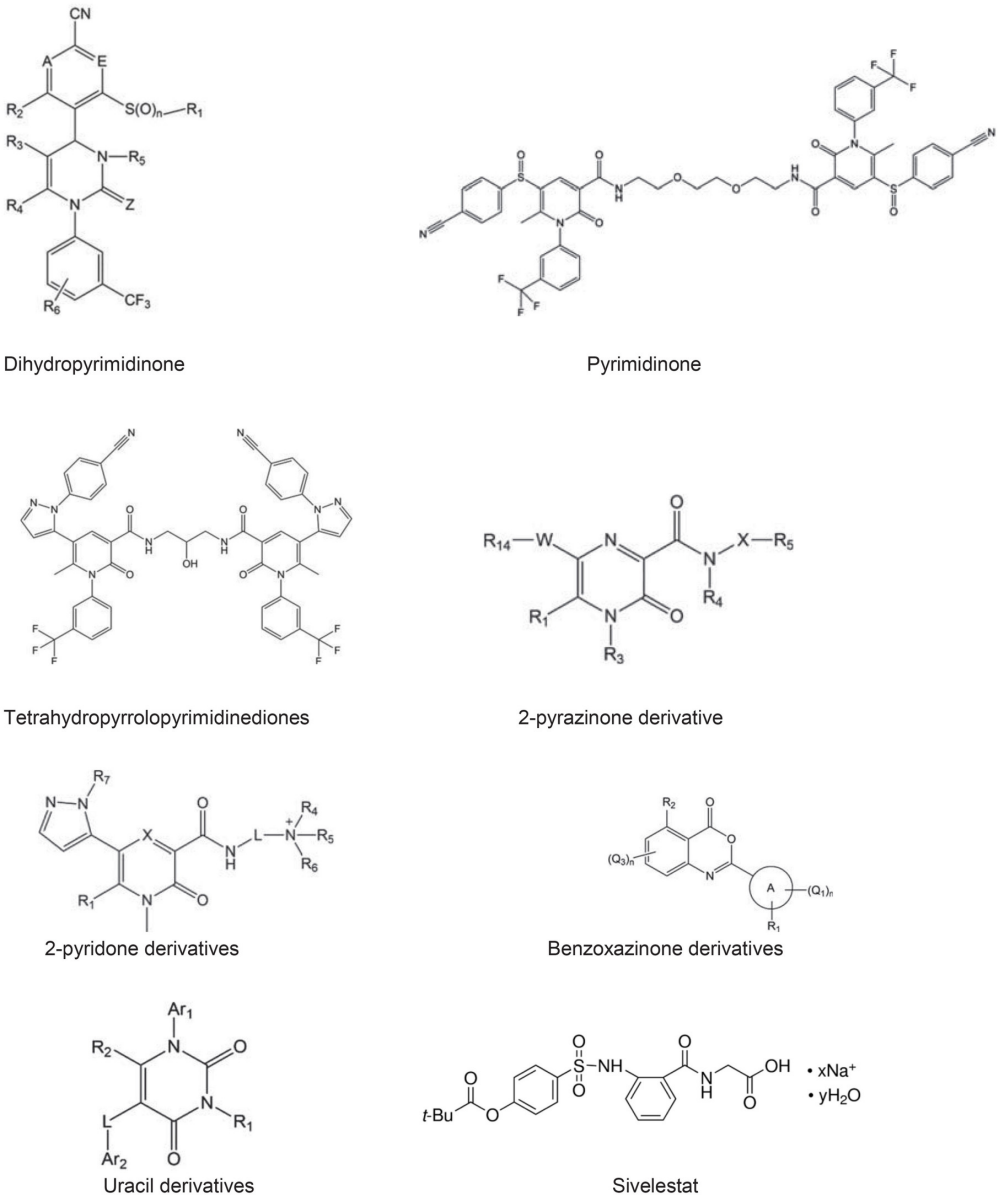
Neutrophil elastase based inhibitors**;** Pyrimidinone, Pyrimidinedione, and Pyrimidine derivative**s** are act as NE inhibitors. The IC_50_ values of dihydropyrimidinone (0.3 nM), pyrimidinone (0.61 nM), tetra hydro pyrrolo pyrimidinediones (0.20 nM), 2-pyrazinone derivative (0.33 nM), 2-pyridone derivatives (3.5 nM), benzoxazinone derivatives (15 nM), uracil derivatives (3.5 nM) (Gadek and Pacht, 1990; Vandivier et al., 2002; Iba et al., 2006), sivelestat (46 nM), indicate that above derivatives are potential inhibitors (Ainge et al., 2010; Hsieh et al., 2010; Ray et al., 2010; Bergstrom et al., 2011; Von Nussbaum et al., 2012). Abbreviation used in the figures A and E both represent C—R_7_ or one of the two ring members A and E represents N and the other represents C—R_7_, in which R_7_ represents in each case hydrogen, fluorine or chlorine, Z represents O or S, *n* represents the number 0, 1, or 2. W represents a 5-membered heterocyclic ring comprising at least one ring heteroatom selected from nitrogen, oxygen, and sulphur. X represents unsubstituted C1-C2 alkylene, L represents C2-C4 alkylene, wherein A is a 5–10 membered heterocyclyl or heteroaryl ring connected to the benzoxazine core by a carbon atom of the heterocyclyl or heteroaryl ring, Q1, Q2, Q3 are halo, pseudohalo, hydroxy, oxo, thia, nitrile, nitro, formyl etc. Ar1 and Ar2 independently represent a 5- to 6-membered aromatic ring group which may contain 1–3 hetero atoms. In the last structure, x indicates number of sodium ions and y is number of water molecules.

There are peptide based inhibitors have been developed against NE, which tend to inhibit NE activity (Iba et al., 2006; Wada et al., 2006). The inhibitory concentrations of these peptides for NE are approximately 0.094 mM, e.g., H-L-Arg-Phg-L-Val-Phg-OH. Comparing with linear peptide, a template fixed beta hairpin peptidomimetics are cyclic peptides, which provide more stability and structural rigidity in physiological conditions duo to their macrolactam cysteine knot scaffold (Kawabata et al., 1991). For example, hPhe-Cys-Thr-Ala-Ser-OctG, Pro-Pro-Gln-Cys-Tyr is a peptide based on fixed beta hairpin with IC_50_ is 6.0 nM, but non-peptide hetrocyclic based inhibitors are more common due to potential target for NE. The heterocyclic NE inhibitors are small molecular mass, structurally diverse and act as reversible competitive inhibitors. A pyrimidine moiety with significant characteristic of heterocyclic nitrogen, believe to be potential target for NE, which is applied for pharmaceutical designs (Kawabata et al., 1991). Since pyrimidine derivatives exhibit therapeutic properties such as, antifungal, calcium-blocking and anti-inflammatory activities, they are also been used to inhibit NE activity. The other class of hetrocyclic NE inhibitor is dihydropyrimidinones, which act as calcium channel modulators and antagonist of α 1A adrenoreceptor. The derivatives of these compounds have been found to be potent NE inhibitors, therefore proposed as potential therapeutics for COPD (Hansen et al., 2011; Von Nussbaum et al., 2012). Study suggested that dihydropyrimidinone derivatives are claimed to be potential NE inhibitors and IC_50_ value for most of these compounds are in the range of pM to nM (Hansen et al., 2011). Pyrimidinone, pyrimidinedione, and pyrimidine derivatives were revealed to have IC_50_ values for NE in the ranges of 0.2–1.0 nM (Figure 4).

The dihydropyrimidinones groups of inhibitor function as calcium channel modulators as well as selective α 1a-adrenoceptor antagonist. Hansen et al. (2011), suggested that the derivatives of these compounds have been found to be potent human NE inhibitors and have thus been proposed as potential COPD therapeutics (Kawabata et al., 1991). Using these inhibitors, NE selectivity assays showed selectivity ranging from 1- to > 300-fold for various proteases and demonstrated desirable human NE inhibitory activity in an NE-induced lung hemorrhage model in rats, and there has been no noticeable side effect reported in these study (Kawabata et al., 1991). The protective effects of these compounds were also confirmed in human NE and lipopolysaccharide (LPS) induced rat lung injury models (Hsieh et al., 2010). The tetrahydropyrrolo- pyrimidinediones display human NE inhibitory activity in the range of 1–50 nM, and they were shown to protect rat lungs in a human NE-induced acute lung injury model (Bergstrom et al., 2011). 2-pyrazinones derivative displayed IC_50_ values for human NE activity in the nano molar range. These compounds generally administered orally or as dry powder for inhalination without any noticeable side effect (Ray et al., 2010). A benzoxazinone groups exhibited inhibitory activity on human NE in the <15–150 nM range. The beneficial effect of benzoxazinone was tested in a rat LPS induced acute lung injury model, a heterocyclic acylating moiety of those compounds inhibited human NE via a mechanism involving the formation of an acyl enzyme intermediate (Hsieh et al., 2010).

The current information suggests that Sivelestat (Figure 4) is a NE inhibitor approved in Japan and South Korea for acute lung injury, acute respiratory distress syndrome in patients with systemic inflammatory response. In non-clinical studies, Sivelestat seems to show benefit in lung injury without inhibiting the host immune defense. The available evidence suggests that Sivelestat may show some benefit in the treatment of acute lung injury/acute respiratory distress syndrome, but large and randomized controlled trials are need to be done in specific pathophysiological conditions to explore the potential benefits in case of COPD.

## Summary and future direction

Many proteases are involved in the inflammatory process of COPD and responsible for the destruction of elastin fibers in the lung parenchyma, therefore, identification of those enzymes and other inflammatory mediators as well as understanding their interactions are important for the development of anti-inflammatory treatments for this disease. Redundancy among protease functions and the multiple beneficial effects of MMPs at different sites make those classes of proteases a challenging task to use in the field. Despite of published evidences there are challenges to consider the complexity of proteases and their inhibitors networks. No single molecule could be proposed yet for wide use in clinical practice, therefore, there is a need of basic study to look into detail with modern tools of biochemistry and molecular biology. The current approach of targeting specific pathways/ or processes of these proteases, but only after detail evaluation and the mechanism of these targets are likely to be a safer and more practical approach. The invention of novel NE inhibitors with high potency and low toxicity are the requirement of present time as in the future it may be used for patient therapy. To find out the cause of destruction of lung tissue in patients, it would be worth to study the role of proteases and their imbalance between proteolytic and anti-proteolytic activities and related oxidative stress in great details. The clinical diagnosis of COPD cases could be strengthened by adopting translational bench-to-bedside approach applying proteases biochemistry to biological samples obtained from clinically diagnosed patients. The complexity of COPD will definitely require a multi-target therapeutic approach. Therefore, other targets beside proteases would be beneficial to consider in COPD.

## Author contributions

KP is an expert in Protease Biochemistry and generate ideas and wrote the review, SD is an expert in Pulmonary Medicine and help in writing process, and PM is an expert in Environmental toxicology and help in writing process.

### Conflict of interest statement

The authors declare that the research was conducted in the absence of any commercial or financial relationships that could be construed as a potential conflict of interest.
